# Delicate, Backward, Puny, and Stunted Children

**Published:** 1896-01

**Authors:** 


DELICATE, BACKWARD, PUNY, AND STUNTED CHILDREN: Their developmental defects, and physical, mental, and moral peculiarities considered as ailments, amenable to treatment by medicines. By J. Compton Burnett, M. D. Philadelphia: Bœricke & Tafel, 1011 Arch Street. 1896. Price, cloth, $1; by mail, $1.05.

We quote from the preface the better to set forth the motive of the book: "In his daily work the practical physician meets with a number of abnormal states that are not readily classified. * * * We say of certain children that they are delicate, backward, peculiar, odd, stunted, puny, and the like, without being able exactly to state what disease they are suffering from.

"The development of a given child receives a shock from a fall or fright; or its further growth is arrested by some acute disease such as measles or influenza; or a child is glum, taciturn, excitable, or what not, and yet people hardly know what is wrong or how to set about putting the wrong right. Again, some children do not see, hear, or speak properly; or they are unclean in their habits, wet their clothes or their beds, and cannot be taught nice, sweet ways like their fellows. This little work is intended to show that such abnormalities depend upon physical conditions, that can be put right by properly chosen remedies, and in no other way so well."

In accordance with the object thus announced the author proceeds to give the best homœopathic treatment for such cases, and to illustrate by recounting from his clinical records, his own brilliant successes in this line.

There are few of the profession who have not read the previous monographs of Dr. Burnett, with which he has favored the profession from time to time.

The present work is just as graceful, just as clear, just as convincing, and helpful as his other books on correlated subjects, and we coadially recommend it.

